# Gender differences in the relationships between housework and metabolic markers: a longitudinal cohort study in China

**DOI:** 10.1186/s12889-022-12566-6

**Published:** 2022-02-17

**Authors:** Xiao-qin Wang, Xiao-han Ren, Wen-jing Kou, Yang Li, Zhao-zhao Hui, Jia-ru Sun, Ming-xu Wang

**Affiliations:** 1 Health Science Center, School of Public Health, Xi’an Jiaotong University, 76 Yanta West Road, Xi’an, 710061 China; 2 Department of Nursing, Air Force Medical University, Xi’an, China

**Keywords:** Housework, Metabolic Syndrome, Stress, Gender Differences

## Abstract

**Background:**

Metabolic syndrome has become a major health threat throughout the world, but there are few studies that focus on the effects of housework on human metabolism. This study explores the association between housework and metabolic markers and examines whether there are gender differences in the relationship of housework intensity on these markers.

**Methods:**

We obtained data for 2,624 participants from the China Health and Nutrition Survey and used binary logistic regression to analyze the association between housework and metabolic markers (triglycerides, high- and low-density lipoprotein cholesterol, hemoglobin, blood glucose, cholesterol, and blood pressure).

**Results:**

We observed no association between housework and metabolic markers for men. However, we find that women who engaged in housework had a higher risk of triglycerides than those who did not (OR=1.16, 95% CI: 1.16, 4.25). Compared with low-intensity, we also find that women who performed moderate- and high-housework intensity had a higher risk of triglycerides (moderate-intensity: OR=1.78, 95% CI: 1.14, 2.78; high-intensity: OR=1.91, 95% CI: 1.22, 2.98), MetS (OR=1.54, 95% CI: 0.98, 2.43; OR=1.68, 95% CI: 1.07, 2.66), pre-hypertension (OR=1.68, 95% CI: 1.08, 2.62; OR=1.63, 95% CI: 1.04, 2.55), and obesity (OR=1.65, 95% CI: 1.01, 2.70; OR=1.66, 95% CI: 1.01, 2.72).

**Conclusion:**

In women, we find that housework is positively associated with the metabolic markers, triglycerides, MetS, and pre-hypertension. However, we did not find evidence that this relationship exists in men, f or any biomarkers we considered. One possible explanation is that people who engage in high-intensity housework are more stressed and sleep less, which could be a mechanism by which housework becomes associated with metabolic disease.

**Supplementary Information:**

The online version contains supplementary material available at 10.1186/s12889-022-12566-6.

## Background

Metabolic syndrome (MetS) is a common metabolic disorder defined by the WHO as a multifaceted continuum of metabolic disorders characterized by obesity, insulin resistance, hypertension, and hyperlipidemia [1, 2]. The incidence of MetS globally ranged from 12.2% to 43.7% over the last ten years, and researchers anticipate that it will continuously increase in the next 10 years [3–5]. In China, the prevalence of MetS among Chinese adults was about 21.3% [3, 4]. Previous evidence has shown that metabolic syndrome increases the risk of type 2 diabetes by a factor of 5 and cardiovascular disease by a factor of 2 [6]. Furthermore, people with MetS spend 60% more on health care costs than the general population [4].

Recently, some studies have demonstrated that housework can influence individuals’ mental and physical health [7, 8]. Heavy housework can not only increase the risk of experiencing mental illness (e.g., depression and suicide) [8, 9], but can also lead to poor physical health outcomes (e.g., chronic lower back disease, upper limb strain, and functional somatic symptoms) [10–12]. Housework may affect health through physiological stress mechanisms and resultant amounts of sleep. Researchers have suggested that people who spend more time doing housework have higher salivary cortisol levels [13]. Cortisol might further affect the body’s fat metabolism by binding to glucocorticoid receptors and activating fat cells [14]. Moreover, the circadian rhythm disorder caused by a lack of sleep can affect people’s metabolism as well. Therefore, housework may indeed have an impact on metabolism, but the current research on the relationship between housework and metabolic markers is limited with no insight into the underlying mechanism of this relationship.

Existing research suggests that housework has different health effects on men and women [7, 12], and different intensities of housework may have different effects as well, regardless of gender. On average, an overburden of housework has been related to the self-reported poor health for women, while a large amount of housework has been related to reduced risks of mortality and sickness among men [15]. In addition, a number of studies have shown that people doing moderate housework have better physical health [7]. Currently, there is no consensus on the impact of housework intensity on health, and there are few reports on objective health indicators as they relate to housework.

Housework is an indispensable part of daily life, and women do most of the housework in most countries around the world [16, 17], especially in China. In China, influenced by traditional Chinese culture’s expectations of them, women undertake more housework work than men. Furthermore, studies have found that the burden of housework increases with a higher number of family members for whom to care [18]. This bears examining because with rapid population growth and China’s current two-child policy, the number of people in need of care is expected to increase significantly in the next few decades.

Although previous studies have demonstrated the association between housework and subjective health, the underlying mechanism remains unclear. Researchers hypothesize that housework can influence subjective health by metabolism and that there are gender differences between these associations. Accordingly, this study examines the associations between housework and metabolic markers and explores whether there are gender differences in these associations by analyzing a large longitudinal cohort study from China.

## Methods

### Study design and data source

This study uses national longitudinal data from the CHNS (China Health and Nutrition Survey), an ongoing, large-scale, longitudinal survey. The study is being conducted by the Carolina Population Center at the University of North Carolina at Chapel Hill (UNC-CH) and the National Institute of Nutrition and Food Safety at the Chinese Center for Disease Control and Prevention (INFS-CCDC). The survey uses a multistage, stratified, random cluster process to select samples in 15 provinces that vary in demography, geography, economic development, and medical resources [19]. The study uses the individual Master-Time Allocation (TIMEA) survey, which provides an opportunity to assess the health and behavior of Chinese people who participate in housework.

Because the data format was different in early years, our sample from it only uses three waves of data from 2009 to 2015. We include the participants aged 18-80 who provided answers in three waves. According to inclusion criteria, subjects were restricted to providing complete baseline information including age, gender, education, employment status, residence, marital status, medical insurance, drinking, and smoking information. If the subject lacked data of any variable, he would be excluded from this study. A total of 3,079 subjects (male=720, female=2,359) were initially included, but 437 were excluded due to the lack of biological data, resulting in a final sample of 2,642. The CHNS has been reviewed and approved by the institutional review committees of the UNC-CH and the INFS-CCDC. Written informed consent was obtained from each participant.

## Measures

### Metabolic markers

The health assessment from the survey measured 9 metabolic markers, including triglycerides, low- and high- density lipoprotein cholesterol (LDL and HDL, respectively), hemoglobin-A1c (HbA1C), fasting blood glucose, cholesterol, blood pressure, obesity, and waist circumference. Biomarker blood data was collected via venipuncture and detected immediately for fasting glucose. Plasma and serum samples were then frozen and stored at -86 °C for laboratory analysis. All samples were analyzed at the national central laboratory in Beijing and strict quality control measures were carried out. Levels of biomarkers were categorized using cut-off points recommended by the International Diabetes Federation, with separate cut-off points for men and women where appropriate (Table 1).

**Table 1:** Definitions of risk biomarker indicators

Biomarker	Definition
High triglycerides	≥150 mg/dL
High LDL	>130 mg/dL
Low HDL	Men: <40 mg/dL; women<50 mg/dL
High HbA1C	≥6.5 %
High glucose	≥ 100 mg/dL
High total cholesterol	≥200 mg/dL
Hypertension	SBP ≥ 140 mmHg or DBP ≥ 90 mmHg or taking antihypertensive drugs
Pre-hypertension	SBP ≥ 130 mmHg AND <140 mmHg or DBP ≥ 85 mmHg AND < 90mmHg or taking antihypertensive drugs
Overweight	BMI ≥ 25 kg/m^2^
Waist circumference	Men’s waist ≥ 90 cm; women’s waist ≥ 80 cm
MetS	1) men’s waist ≥ 90 cm, women’s waist ≥ 80 cm; 2) triglyceride ≥ 150 mg/dL; 3) HDL < 50 mg/dL; 4) blood pressure ≥ 130/85 mmHg or currently used antihypertensive drugs; 5) fasting blood glucose ≥100 mg/dL.

According to the American Heart Association, blood pressure should be correctly measured at least twice when the person being measured has been sitting for a long time. After participants rested for 10 minutes, systolic blood pressure (SBP) and diastolic blood pressure (DBP) were measured three times by a professional using a mercury sphygmomanometer according to the standard procedures [20]. Hypertension is defined as SBP ≥ 140 mmHg or DBP ≥ 90 mmHg, or as the use of antihypertensive drugs. Pre-hypertension is defined as SBP ≥ 130 mmHg and <140 mmHg, or DBP ≥ 85 mmHg and < 90mmHg, or as the use of antihypertensive drugs.

Height was measured without shoes to the nearest 0.1 cm using a portable SECA electronic multifunctional scale, and weight was measured without shoes and in light clothing to the nearest 0.1 kg on a calibrated beam balance. Body mass index (BMI) was calculated as body weight divided by the square of height. Obesity is defined as BMI ≥ 25 kg/m^2^ according to the standards for Chinese people [21].

According to the latest National Cholesterol Education Program Adult Treatment Panel III (NCEP-ATPIII) guidelines for the Chinese population, MetS is defined as having any three or more of the following five components: men’s waist circumference ≥ 90 cm, women’s waist circumference ≥ 80 cm; triglyceride ≥ 150 mg/dL; HDL < 50 mg/dL; pre-hypertension ≥ 130/85 mmHg or current antihypertensive drug use; and fasting blood glucose ≥ 100 mg/dL [22].

### Housework and housework intensity

Participants in the study were asked eight questions about their involvement in housework:

1. Do you buy food for your family?
2. How long does buying food take on average each day?
3. Do you cook for your family?
4. How long does cooking take on average each day?
5. Do you wash and iron clothes?
6. How long does it take on average each day?
7. Do you clean the rooms of your domicile?
8. How long does it take on average each day?

The time spent on each type of housework was summed, and the results were divided into three categories: low-intensity housework (less than 50 minutes/day), moderate-intensity housework (50-180 minutes/day), and high-intensity housework (more than 180 minutes/day).

### Covariates

We identified the covariates of interest for this study by referencing existing research [23, 24]. Age and gender are the important factors that affect metabolism, but socioeconomic factors (such as educational level and economic factors) and individuals’ health behaviors can also affect metabolism through influencing individual health. For this reason, our covariates include gender (male/female) and age (18-40 years, 41-65 years, and ≥ 65 years) as well as socioeconomic variables including education (primary/below, secondary school, tertiary school/above), employment status (yes/no), residence location (rural/urban), marital status (unmarried/divorced/widowed, married/cohabiting with spouse), healthcare insurance (yes/no), drinking (yes/no), and smoking (yes/no) [22–25].

### Statistical analysis

We calculated descriptive statistics by determining the mean, median, standard deviation and inter quartile range of continuous variables to describe the demographic characteristics of participants in the study. To analyze the distribution of demographic characteristics and biological metabolism differences between men and women, we used the Mann-Whitney test and Pearson’s chi-squared test. In addition, we assessed the associations between housework and metabolic indicators by logistic regression analysis. First, we divided the participants into two groups according to their gender for age-related analysis. Second, all covariates were introduced to investigate the correlation between housework and metabolic markers. All statistical analysis was performed using SPSS 22.0 software (Statistical Package for the Social Sciences for Windows, IBM Corp, Armonk, NY, USA). We consider a two-tailed *P*-value < 0.05 to indicate a statistically significant result.

## Results

Table 2 shows the descriptive statistics for participants on metabolic outcomes and covariates (*n*=2,624). This table shows that women accounted for a larger proportion of participants than men and that women were more common in the middle-age group than men. Women provided more hours per-day of chores than men; 41.7% of women took part in high-intensity housework compared to 6.4% of men. In general, the education level of men was higher than that of women. Most men (59.4%) had a secondary education, but nearly half of women (54.1%) had received only primary education. Half of the men (49.9%) lived in cities, while most women (65.9%) lived in rural areas.

**Table 2:** Characteristics of the study sample (*n*=2,624)

		Men (*n*=547)		Women(*n*=2077)		*P*
		n/mean/median	%/SD/IQR	n/mean/median	%/SD/IQR	
Age (*n*/%)						<0.001^*^
	18-40	261	47.7	650	31.3	
	41-64	233	42.6	1290	62.1	
	≥65	53	9.7	137	6.6	
Housework						<0.001^*^
	Yes	264	48.3	62	3	
	No	283	51.7	2015	9.7	
Housework intensity (*n*/%)						<0.001^**^
	0-50 min/day	364	66.5	137	6.6	
	50-180min/day	148	27.1	1074	51.7	
	≥ 180 min/day	35	6.4	866	41.7	
Education (n/%)						<0.001^**^
	Primary school and below	132	24.1	1124	54.1	
	Secondary school	325	59.4	833	40.1	
	Tertiary school or above	90	16.5	120	5.8	
Employment status (*n*/%)						<0.001^**^
	No	223	40.8	1096	52.8	
	Yes	324	59.2	981	47.2	
Residence (*n*/%)						<0.001^**^
	Rural	273	49.9	708	34.1	
	Urban	274	50.1	1369	65.9	
Medical insurance (*n*/%)						<0.001^**^
	No	288	52.7	1545	74.4	
	Yes	259	47.3	532	25.6	
Marital status (*n*/%)						<0.001^**^
	Single/Separated/divorced	90	16.5	174	8.4	
	Married/living with partner	457	83.5	1903	91.6	
Smoking (*n*/%)						<0.001^**^
	non-smoking	220	40.2	2018	97.2	
	current smoking	327	59.8	59	2.8	
Drinking (*n*/%)						<0.001^**^
	non-drinking	227	41.5	1915	92.2	
	current drinking	320	58.5	162	7.8	
Housework time (mean/SD)						
	2009 (min/day)	44.0	65.9	165.7	88.4	<0.001^**^
	2011 (min/day)	48.0	70.3	167.6	86.1	<0.001^**^
	2015 (min/day)	61.8	67.9	176.6	114.7	<0.001^**^
Metabolic markers (median/IQR)						
	Triglycerides (mg/dL)	123.1	121.4	112.5	91.2	<0.001^**^
	Cholesterol (mg/dL)	183.3	46.0	186.8	50.0	0.024^*^
	HDL (mg/dL)	48.7	17.8	54.9	17.0	<0.001^**^
	LDL (mg/dL)	111.0	47.6	114.8	46.0	0.010^*^
	Glucose(mg/dL)	92.5	16.6	91.6	15.8	0.114
	HbA1c (%)	5.5	0.6	5.5	0.6	0.480
	Waist circumference	83.0	14.1	79.0	13.6	<0.001^**^
	BMI	22.6	2.3	22.3	4.4	0.299
	SBP mmHg	120.3	20.0	119.3	21.3	0.001^*^
	DBP mmHg	80.0	14.0	78.0	12.7	<0.001^**^

*SD* Standard deviation, *IQR* Inter-quartile range

^*^P<0.05; **P<0.001 (P represents the gender differences).

There were also significant differences in the proportion of medical insurance between men (47.3%) and women (25.6%). Most participants were married and lived with their spouses. In addition, nearly two-thirds of men reported that they were currently smoking and drinking, with both activities’ showing statistically significant gender differences. The triglycerides, glucose levels, waist circumference, and blood pressures of men were higher than those of women. However, women had higher cholesterol on average (men: 183.3 mg/dL, women: 186.8 mg/dL), HDL (men: 148.7 mg/dL, women: 54.9 mg/dL), and LDL (men: 111.0 mg/dL, women: 114.8 mg/dL).

### Gender differences in housework intensity and metabolic markers

In the study sample, in the low-, moderate-, and high-intensity groups, the proportion of men with abnormal metabolic indicators was significantly higher than that of women, especially for triglycerides, HDL, MetS, and waist circumference (Table 3). There were significant differences in MetS and pre-hypertension among men in low-intensity, moderate-intensity, and high-intensity groups. Men who performed high-intensity housework had a significantly higher prevalence of MetS (45.7%) compared to those who performed only moderate-intensity (28.8%) and low-intensity (39.9%) housework. The prevalence of women’s triglycerides in the three housework intensity groups were 20.4%, 30.1%, and 32.8%, respectively, and the risk of MetS was only marginally different among these three groups of women. No significant differences among different housework intensity were observed for LDL, HbA1C, glucose, hypertension, or obesity.

**Table 3:** Gender differences between housework intensity and metabolic markers

	Men				Women				*P*^b^
Total *N* (%)	0-50 min/day	50-180min/day	≥ 180 min/day	*P*^a^	0-50 min/day	50-180min/day	≥ 180 min/day	*P*^a^	
Triglycerides				0.690				0.012^*^	<0.001^**^
Normal	224(61.5)	85(57.4)	21(60.0)		109(95.1)	751(69.9)	582(67.2)		
High	140(38.5)	63(42.6)	14(40.0)		28(20.4)	323(30.1)	284(32.8)		
LDL				0.169				0.234	0.109
Normal	267(73.4)	97(65.5)	23(65.7)		100(73.0)	708(65.9)	585(67.6)		
High	97(26.6)	51(34.5)	12(34.3)		37(27.0)	366(34.1)	281(32.4)		
HDL				0.160				0.846	<0.001^**^
Normal	176(48.4)	58(39.2)	15(42.9)		91(66.4)	711(66.2)	563(65.0)		
Low	188(51.6)	90(60.8)	20(57.1)		46(33.6)	363(33.8)	303(35.0)		
HbA1c				0.739				0.601	0.648
Normal	336(92.3)	136(91.9)	31(88.6)		129(94.2)	1010(94.0)	805(93.0)		
High	28(7.7)	12(8.1)	4(11.4)		8(5.8)	64(6.0)	61(7.0)		
Glucose				0.809				0.814	0.546
Normal	258(70.9)	105(70.9)	23(65.7)		105(76.6)	796(74.1)	643(74.2)		
High	106(29.1)	43(29.1)	12(34.3)		32(23.4)	278(25.9)	223(25.8)		
Cholesterol				0.083				0.374	0.080
Normal	259(71.2)	91(61.5)	22(62.9)		95(69.3)	678(63.1)	558(64.4)		
High	105(28.8)	57(38.5)	13(37.1)		42(30.7)	396(36.9)	308(3.56)		
MetS				0.014^*^				0.066	0.002^*^
No	259(71.2)	89(60.1)	19(54.3)		108(78.8)	779(72.5)	604(69.7)		
Yes	105(28.8)	59(39.9)	16(45.7)		29(21.2)	295(27.5)	262(30.3)		
Hypertension				0.618				0.673	0.488
No	286(78.6)	111(75.0)	26(75.3)		112(81.8)	870(81.0)	689(79.6)		
Yes	78(21.4)	37(25.0)	9(25.7)		25(18.2)	204(19.0)	177(20.4)		
Pre-hypertension and Hypertension				0.046^*^				0.115	0.018^*^
No	240(65.9)	90(60.8)	16(45.7)		103(75.2)	719(66.9)	574(66.3)		
Yes	124(34.1)	58(39.2)	19(54.3)		34(24.8)	355(33.1)	292(33.7)		
Overweight				0.991				0.103	0.214
No	271(74.5)	111(75.0)	26(74.3)		116(84.7)	838(78.0)	663(76.6)		
Yes	93(25.5)	37(25.0)	9(25.7)		21(15.3)	236(22.0)	203(23.4)		
WC				0.053				0.175	<0.001^**^
Normal	274(75.3)	100(67.6)	21(60.0)		77(56.2)	562(52.3)	425(49.1)		
High	90(24.7)	48(32.4)	14(40.0)		60(43.8)	512(47.7)	441(50.9)		

*WC* Waist circumference

^*^P<0.05; **P<0.001. (*P*^a^ represents the difference in housework intensity; *P*^*b*^ represents the gender differences in the same housework category)

### Housework intensity and metabolic markers

#### Men

Table 4 shows the associations between housework intensity and metabolic markers for men. In the age-adjusted model, we find that housework is significantly associated with HDL, cholesterol, and MetS (*P*<0.001). Compared with low-intensity housework, men who performed moderate-intensity housework were 49% more likely to have low HDL (OR = 1.49, 95% CI: 1.01, 2.21). However, after adjusting for all covariates, the associations among HDL, total cholesterol, MetS, and housework disappeared, although the relationship between HDL and housework did weakly remain. No associations were observed between hours spent doing housework per day and metabolic markers for men (the models of other covariates are shown in Additional file 1: Appendix I).

**Table 4:** Age-adjusted and multiple logistic regression models of housework and metabolic markers for Chinese men

	Age-adjusted OR (95%CI)										
	Triglycerides	LDL	HDL	HbA1c	Glucose	Cholesterol	MetS	Hypertension	Pre-hypertension & Hypertension	Overweight	WC
Housework											
No	Ref.	Ref.	Ref.	Ref.	Ref.	Ref.	Ref.	Ref.	Ref.	Ref.	Ref.
Yes	0.97(0.68,1.36)	1.02(0.71,1.49)	1.36(0.97,1.90)^a^	1.07(0.58,2.00)	1.03(0.71,1.49)	1.11(0.77,1.59)	1.26(0.87,1.82)	0.86(0.57,1.31)	1.19(0.83,1.72)	0.87(0.59,1.29)	1.11(0.76,1.62)
Housework intensity											
0-50 min/day	Ref.	Ref.	Ref.	Ref.	Ref.	Ref.	Ref.	Ref.	Ref.	Ref.	Ref.
50-180min/day	1.17(0.79,1.72)	1.32(0.87,2.00)	1.49(1.01,2.21)^b^	0.90(0.44,1.84)	0.89(0.58,1.37)	1.44(0.96,2.17) ^a^	1.44(0.96,2.16) ^a^	0.99(0.62,1.58)	1.03(0.68,1.57)	0.88(0.57,1.38)	1.27(0.83,1.96)
≥ 180 min/day	1.08(0.52,2.22)	1.12(0.53,2.39)	1.32(0.64,2.68)	1.06(0.34,3.31)	0.97(0.45,2.06)	1.25(0.60,2.62)	1.53(0.74,3.16)	0.72(0.31,1.66)	1.37(0.65,2.86)	0.82(0.37,1.86)	1.44(0.69,3.02)
	Fully adjusted OR (95%CI)										
	Triglycerides	LDL	HDL	HbA1c	Glucose	Cholesterol	MetS	Hypertension	Pre-hypertension & Hypertension	Overweight	WC
Housework											
No	Ref.	Ref.	Ref.	Ref.	Ref.	Ref.	Ref.	Ref.	Ref.	Ref.	Ref.
Yes	0.89(0.63,1.27)	1.03(0.70,1.50)	1.36(0.96,1.93)^a^	0.98(0.52,1.88)	0.97(0.66,1.42)	1.05(0.73,1.53)	1.22(0.84,1.78)	0.83(0.54,1.27)	1.15(0.79,1.67)	0.78(0.52,1.17)	1.01(0.68,1.50)
Housework intensity											
0-50 min/day	Ref.	Ref.	Ref.	Ref.	Ref.	Ref.	Ref.	Ref.	Ref.	Ref.	Ref.
50-180min/day	1.10(0.74,1.64)	1.24(0.81,189)	1.43(0.96,2.14)^a^	0.86(0.41,1.79)	0.84(0.54,1.30)	1.36(0.90,2.04)	1.35(0.89,2.05)	1.01(0.63,1.63)	1.05(0.69,1.61)	0.80(0.51,1.27)	1.15(0.74,1.78)
≥ 180 min/day	0.96(0.45,2.02)	1.02(0.48,2.22)	1.11(0.53,2.34)	0.94(0.29,3.07)	1.05(0.48,2.30)	1.08(0.50,2.32)	1.38(0.66,2.92)	0.76(0.32,1.79)	1.40(0.65,2.99)	0.71(0.31,1.65)	1.19(0.56,2.55)

*WC* Waist Circumference, *OR* Odds Ratio, *CI* Confidence Interval, *Ref.* Reference.

a: *p*<0.1; b: *p*<0.05; c: *p*<0.001.

#### Women

Table 5 shows the associations between housework intensity and metabolic markers for women. In the age-adjusted model, women who engaged in housework had a high risk of high triglycerides (OR = 2.07, 95% CI: 1.07, 4.02), and women who performed high-intensity housework were more likely to have high triglycerides, MetS, pre-hypertension, and obesity (respectively, 190%, 169%, 165%, and 166%) compared with women in the low-intensity housework group. After adjusting for other covariates, these associations remained unchanged and even strengthened no matter if they were in the moderate-intensity group or the high intensity group (high triglycerides: moderate-intensity: OR=1.78, 95% CI: 1.14, 2.78, high-intensity: OR=1.91, 95% CI: 1.22, 2.98; MetS: moderate-intensity: OR=1.54, 95% CI: 0.98, 2.43, high-intensity: OR=1.91, 95% CI: 1.07, 2.66; pre-hypertension: moderate-intensity: OR=1.68, 95% CI: 1.08, 2.62, high-intensity: OR=1.63, 95% CI: 1.04, 2.55; obesity: moderate-intensity: OR=1.65, 95% CI: 1.01, 2.70, high-intensity: OR=1.66, 95% CI:1.01, 2.72). We find significant correlation between housework intensity and high LDL, low HDL, high HbA1C, high glucose, hypertension, and waist circumference for women (the models of other covariates are shown in Additional file 1: Appendix II).

**Table 5:** Age-adjusted and multiple logistic regression models of housework and metabolic markers for Chinese women.

	Age-adjusted OR (95%CI)										
	Triglycerides	LDL	HDL	HbA1c	Glucose	Cholesterol	MetS	Hypertension	Pre-hypertension & Hypertension	Overweight	WC
Housework											
No	Ref.	Ref.	Ref.	Ref.	Ref.	Ref.	Ref.	Ref.	Ref.	Ref.	Ref.
Yes	2.07(1.07,4.02) ^b^	1.42(0.78,2.58)	1.18(0.69,2.05)	1.42(0.43,4.69)	0.97(0.54,1.76)	1.50(0.84,2.71)	1.33(0.71,2.51)	1.01(0.52,1.96)	1.06(0.60,1.95)	1.93(0.91,4.11) ^a^	1.06(0.63,1.77)
Housework intensity									0.787		
0-50 min/day	Ref.	Ref.	Ref.	Ref.	Ref.	Ref.	Ref.	Ref.	Ref.	Ref.	Ref.
50-180min/day	1.69(1.09,2.64) ^b^	1.43(0.94,2.17) ^a^	1.01(0.70,1.47)	1.14(0.52,2.49)	1.15(0.75,1.77)	1.35(0.90,2.03)	1.47(0.93,2.31) ^a^	1.11(0.68,1.81)	1.65(1.05,2.57) ^b^	1.56(0.96,2.54) ^a^	1.16(0.80,1.67)
≥ 180 min/day	1.90(1.22,2.98) ^c^	1.31(0.86,1.99)	1.06(0.73,1.55)	1.36(0.62,2.97)	1.13(0.73,1.75)	1.25(0.83,1.83)	1.69(1.07,2.67) ^b^	1.22(0.75,2.00)	1.65(1.06,2.59) ^b^	1.66(1.02,2.72) ^b^	1.29(0.89,1.87)
Fully-adjusted OR (95%CI)											
	Triglycerides	LDL	HDL	HbA1c	Glucose	Cholesterol	MetS	Hypertension	Pre-hypertension & Hypertension	Overweight	WC
Housework											
No	Ref.	Ref.	Ref.	Ref.	Ref.	Ref.	Ref.	Ref.	Ref.	Ref.	Ref.
Yes	2.18(1.16,4.25) ^b^	1.44(0.80,2.62)	1.18(0.68,2.05)	1.40(0.42,4.65)	0.96(0.53,1.74)	1.53(0.84,2.77)	1.41(0.75,2.63)	0.98(0.50,1.92)	1.07(0.59,1.94)	1.95(0.92,4.16) ^a^	1.07(0.64,1.81)
Housework intensity											
0-50 min/day	Ref.	Ref.	Ref.	Ref.	Ref.	Ref.	Ref.	Ref.	Ref.	Ref.	Ref.
50-180min/day	1.78(1.14,2.78) ^b^	1.47(0.97,2.23) ^a^	1.03(0.71,1.51)	1.19(0.55,2.60)	1.15(0.75,1.77)	1.38(0.92,2.07)	1.54(0.98,2.43) ^a^	1.13(0.70,1.84)	1.68(1.08,2.62) ^b^	1.65(1.01,2.70) ^b^	1.21(0.84,1.76)
≥ 180 min/day	1.91(1.22,2.98)^c^	1.30(0.85,1.98)	1.05(0.71,1.54)	1.30(0.59,2.84)	1.13(0.73,1.75)	1.24(0.82,1.88)	1.68(1.07,2.66) ^b^	1.20(0.73,1.96)	1.63(1.04,2.55) ^b^	1.66(1.01,2.72) ^b^	1.29(0.90,1.88)

*WC* Waist Circumference, *OR* Odds Ratio, *CI* Confidence Interval, *Ref.* Reference.

a: *p*<0.1; b: *p*<0.05; c: *p*<0.001.

## Discussion

This study finds that housework is associated with metabolic risk and that the risk increases with housework intensity, especially for women. Compared with low-intensity housework, women in the moderate- and high-intensity housework groups had a higher risk of high triglycerides, MetS, pre-hypertension, and obesity in our age-adjusted model. Furthermore, after adjusting for covariates, associations for women were strengthened. In this study, housework seems to have little impact on metabolic indicators for men; we observed only a slight relationship between HDL and MetS and housework intensity for men. These findings are similar to the results of previous studies[15, 26]; people engaged in more hours of housework everyday have reported poorer health than those engaged in low-intensity housework. In addition, this study finds that more women undertook high-intensity housework in China, which is consistent with previous studies on the gender distribution of housework in China [17].

Consistent with prior results, this study indicates that age is a positive predictor of MetS [27], and we also find that the prevalence of MetS may also vary depending on the residence, lifestyle, and cultural behavior [25]. This study suggests that compared with low and moderate housework intensity, high housework intensity is associated with metabolic risk for women, which is consistent with the results of prior research on the relationship between parental caregiving and metabolism, suggesting that moderate and high intensity housework may be potential influencing factors for metabolic risk [24]. However, Brooks et al. found that housework can contribute to the 30 minutes per day of moderate-intensity activity that can confer health benefits [28]. Therefore, housework intensity may be an important factor that affects women’s metabolic function. There are several potential mechanisms that may affect metabolic risk through housework intensity, and we discuss each briefly below.

First, Chronic stress may be a mechanism related to housework intensity and metabolic risk. Owoo et al. found that women who performed high-intensity housework often experienced stress and illness [29]. Chronic stress is a process of mutual induction and activation between the hypothalamic-pituitary-adrenal axis (HPA) and the reninangiotensin-aldosterone system (RAAS) [30, 31]. High activation of HPA is associated with hyper-glucocorticoids and subclinical systemic inflammation and can destroy the metabolism of muscle mass and affect skeletal muscle mitochondrial function and cause metabolic disease [30]. However, excessive activation of the HPA not only directly affects adipose tissue but also causes changes in eating behavior [32]. When perceived stress or real chronic stressors exist, diet control can be lost as eating can be a pleasure reward for the anti-adaptation of stress [33]. In addition, long-term chronic stress can lead to the hypertrophy and proliferation of adipocytes, change the secretion of adipokine, and cause the attraction and activation of interstitial adipose immune cells [34].

The second mechanism may be a lack of sleep. Pepin found that women engaged in housework sleep less because they spend more time doing housework [35]. The circadian rhythm disturbances caused by reduced sleep time can adversely affect their metabolisms [36]. Finally, household air pollution may be a potential mechanism. Studies have shown that free radicals produced by cooking can induced lipid peroxidation, leading to abnormal changes in regular blood lipids and affecting women’s MetS and cholesterol [37].

For men, we find that housework intensity is not associated with men’s metabolic markers. However, Chinese men in general spend less time doing housework due to the influence of traditional Chinese culture, and the number of men in our study was limited. Differently from women, housework may increase men’s daily activity levels and thus have a beneficial effect. Most men may not have sufficient physical activity, and participating in housework provides an opportunity for them to achieve a certain amount of physical activity, which can produce health benefits and reduce the risk of cardiovascular disease [23]. In addition, housework can improve men’s mental health. Some studies suggest that men are happier and have lower levels of psychological stress when doing housework [15, 38]. Furthermore, men’s drinking and smoking behavior can affect the secretion and metabolism of certain hormones [39, 40], thus overshadowing the effect of housework on their metabolisms. The underlying mechanism between male housework and metabolism remains to be pinned down precisely, but it may be that there are several competing mechanisms.

This study has several advantages over previous work. First, we use a large regional sample. Second, health information in this study was obtained under fasting and standard laboratory conditions. In most previous studies, the assessment of participants’ health status was based on self-reported health outcomes, rather than objective data. Finally, this study investigated associations between housework and metabolic markers and provided evidence for the effects of housework on metabolism for both men and women. However, further study to explore the mechanisms of these effects is needed.

Nevertheless, this study has some limitations. First, the data for time spent on housework were self-reported, which may introduce recall bias into the sample. For example, most people who cook tend to ignore the preparation time before cooking and the cleaning time after cooking, and some people who do less housework will exaggerate the time they spend cooking to cover up the fact that they don’t do housework. Second, some female participants in this study may be in menopause, which produces changes in estrogen that are related to the rise of HDL, blood sugar, triglycerides and others markers [41]. Third, the effect of housework on metabolism risk may be affected by selection bias. The healthier body a participant had, the more housework s/he could take up. Finally, the sample size of men is small, which may affect the accuracy of the results. Therefore, the results of this study need to be carefully interpreted and could benefit from a larger sample size in future studies.

## Conclusion

This study shows that housework is associated with metabolic risk, especially among Chinese women. We find that women who engage in moderate- and high-intensity housework have higher risk of high triglycerides, MetS, pre-hypertension, and obesity compared to those who engage only in low-intensity housework. After adjusting for covariates, we find that all these associations for women were strengthened. However, further research is needed to explore the mechanisms of housework and metabolic risk.

## Statement

All methods were performed in accordance with the relevant guidelines and regulations.

## Supplementary Information

**Additional file 1: Appendix I.** Single covariate adjusted binary logistic regression models of housework and metabolic markers for Chinese men. **Appendix II.** Single covariate adjusted binary logistic regression models of housework and metabolic markers for Chinese women.

## Data Availability

The data that support the conclusions of this article are available from the China Health and Nutrition Survey repository, https://www.cpc.unc.edu/projects/china.
