# Supplementary material for: Gender differences in the relationships between housework and metabolic markers: a longitudinal cohort study in China
Source: BMC Public Health. 2022 Feb 17;22:336. doi: 10.1186/s12889-022-12566-6 (PMC8851696; doi:10.1186/s12889-022-12566-6)
Supplement: Supplementary file 1 — Additional file 1: Appendix I. Single covariate adjusted binary logistic regression models of housework and metabolic markers for Chinese men. Appendix II. Single covariate adjusted binary logistic regression models of housework and metabolic markers for Chinese women. [file 12889_2022_12566_MOESM1_ESM.docx]

Appendix I Single covariate adjusted binary logistic regression models of housework and metabolic markers for Chinese men.

|  | Education-adjusted OR (95%CI) | | | | | | | | | | |
| --- | --- | --- | --- | --- | --- | --- | --- | --- | --- | --- | --- |
|  | Triglycerides | LDL | HDL | HbA1c | Glucose | Cholesterol | MetS | Hypertension | Pre-hypertension & Hypertension | Overweight | WC |
| Housework |  |  |  |  |  |  |  |  |  |  |  |
| No | Ref. | Ref. | Ref. | Ref. | Ref. | Ref. | Ref. | Ref. | Ref. | Ref. | Ref. |
| Yes | 0.96(0.68,1.35) | 1.08(0.75,1.57) | 1.35(0.96,1.89)^a^ | 1.13(0.61,2.10) | 1.06(0.74,1.53) | 1.12(0.78,1.61) | 1.39(0.97,2.00)^a^ | 0.99(0.66,1.48) | 1.33(0.94,1.89) | 0.89(0.61,1.32) | 1.18(0.81,1.72) |
| Housework intensity | | | | | | | | | | | |
| 0-50 min/day | Ref. | Ref. | Ref. | Ref. | Ref. | Ref. | Ref. | Ref. | Ref. | Ref. | Ref. |
| 50-180min/day | 1.16(0.79,1.71) | 1.43(0.95,2.16)^a^ | 1.41(0.95,2.09)^a^ | 1.07(0.53,2.18) | 1.00(0.66,1.53) | 1.54(1.03,2.30)^a^ | 1.61(1.08,2.41)^b^ | 1.26(0.80,1.97) | 1.27(0.86,1.89) | 0.94(0.61,1.47) | 1.42(0.94,2.17)^a^ |
| ≥ 180 min/day | 1.00(0.49,2.04) | 1.39(0.66,2.91) | 1.13(0.56,2.30) | 1.61(0.53,4.93) | 1.30(0.62,2.71) | 1.44(0.70,2.98) | 1.99(0.98,4.03)^a^ | 1.37(0.61,3.06) | 2.44(1.21,4.94)^b^ | 0.93(0.42,2.07) | 1.89(0.92,3.89)^a^ |
|  | Employment-adjusted OR (95%CI) | | | | | | | | | | |
|  | Triglycerides | LDL | HDL | HbA1c | Glucose | Cholesterol | MetS | Hypertension | Pre-hypertension & Hypertension | Overweight | WC |
| Housework |  |  |  |  |  |  |  |  |  |  |  |
| No | Ref. | Ref. | Ref. | Ref. | Ref. | Ref. | Ref. | Ref. | Ref. | Ref. | Ref. |
| Yes | 0.97(0.69,1.37) | 1.07(0.74,1.55) | 1.36(0.97,1.91)^a^ | 1.12(0.61,2.09) | 1.07(0.74,1.55) | 1.10(0.76,1.58) | 1.39(0.97,1.99)^a^ | 0.97(0.65,1.45) | 1.30(0.91,1.85) | 0.90 (0.61,1.32)^a^ | 1.16(0.79,1.69) |
| Housework intensity | | | | | | | | | | | |
| 0-50 min/day | Ref. | Ref. | Ref. | Ref. | Ref. | Ref. | Ref. | Ref. | Ref. | Ref. | Ref. |
| 50-180min/day | 1.20(0.81,1.77) | 1.43(0.95,2.15)^a^ | 1.48(1.00,2.19)^b^ | 1.05(0.52,2.14) | 1.01(0.66,1.54) | 1.50(1.00,2.25)^b^ | 1.64(1.10,2.45)^b^ | 1.18(0.75,1.86) | 1.21(0.81,1.80) | 0.98(0.63,1.52) | 1.44(0.95,2.19)^a^ |
| ≥ 180 min/day | 1.12(0.55,2.30) | 1.34(0.64,2.83) | 1.36(0.67,2.77) | 1.51(0.49,4.68) | 1.35(0.64,2.85) | 1.27(0.61,2.65) | 2.11(1.04,4.31)^b^ | 1.10(0.49,2.46) | 2.00(0.98,4.07)^a^ | 1.03(0.46,2.30) | 1.91(0.92,3.95)^a^ |
|  | Residence-adjusted OR (95%CI) | | | | | | | | | | |
|  | Triglycerides | LDL | HDL | HbA1c | Glucose | Cholesterol | MetS | Hypertension | Pre-hypertension & Hypertension | Overweight | WC |
| Housework |  |  |  |  |  |  |  |  |  |  |  |
| No | Ref. | Ref. | Ref. | Ref. | Ref. | Ref. | Ref. | Ref. | Ref. | Ref. | Ref. |
| Yes | 0.94(0.67,1.33) | 1.04(0.72,1.51) | 1.30(0.93,1.83) | 1.06(0.57,1.98) | 1.01(0.70,1.48) | 1.09(0.76,1.57) | 1.34(0.93,1.93) | 0.97(0.65,1.45) | 1.30(0.92,1.85) | 0.87(0.59,1.28) | 1.12(0.77,1.64) |
| Housework intensity | | | | | | | | | | | |
| 0-50 min/day | Ref. | Ref. | Ref. | Ref. | Ref. | Ref. | Ref. | Ref. | Ref. | Ref. | Ref. |
| 50-180min/day | 1.18(0.80,1.73) | 1.44(0.96,2.18)^a^ | 1.45(0.98,2.14)^a^ | 0.91(0.45,1.87) | 0.97(0.64,1.49) | 1.54(1.03,2.30)^a^ | 1.60(1.07,2.40)^b^ | 1.20(0.76,1.88) | 1.23(0.83,1.82) | 0.94(0.60,1.47) | 1.43(0.94,2.18)^a^ |
| ≥ 180 min/day | 1.05(0.52,2.13) | 1.43(0.69,2.99) | 1.25(0.62,2.51) | 1.14(0.37,3.54) | 1.22(0.58,2.55) | 1.45(0.70,2.99) | 2.00(0.99,4.06)^a^ | 1.23(0.55,2.73) | 2.24(1.11,4.52)^b^ | 0.96(0.43,2.13) | 1.96(0.95,4.03)^a^ |
|  | Medical insurance-adjusted OR (95%CI) | | | | | | | | | | |
|  | Triglycerides | LDL | HDL | HbA1c | Glucose | Cholesterol | MetS | Hypertension | Pre-hypertension & Hypertension | Overweight | WC |
| Housework |  |  |  |  |  |  |  |  |  |  |  |
| No | Ref. | Ref. | Ref. | Ref. | Ref. | Ref. | Ref. | Ref. | Ref. | Ref. | Ref. |
| Yes | 0.92(0.65,1.30) | 1.07(0.74,1.55) | 1.29(0.92,1.81) | 1.08(0.58,2.01) | 1.03(0.71,1.49) | 1.13(0.78,1.62) | 1.30(0.90,1.87) | 0.91(0.61,1.37) | 1.24(0.87,1.77) | 0.85(0.58,1.26) | 1.09(0.75,1.60) |
| Housework intensity | | | | | | | | | | | |
| 0-50 min/day | Ref. | Ref. | Ref. | Ref. | Ref. | Ref. | Ref. | Ref. | Ref. | Ref. | Ref. |
| 50-180min/day | 1.17(0.80,1.72) | 1.44(0.96,2.18)^a^ | 1.43(0.97,2.11)^a^ | 1.04(0.51,2.10) | 0.98(0.64,1.50) | 1.55(1.04,2.31)^b^ | 1.60(1.07,2.40)^b^ | 1.18(0.75,1.86) | 1.21(0.81,1.81) | 0.95(0.61,1.48) | 1.43(0.94,2.18)^a^ |
| ≥ 180 min/day | 0.96(0.47,1.97) | 1.40(0.67,2.95) | 1.13(0.55,2.29) | 1.38(0.45,4.26) | 1.17(0.56,2.47) | 1.47(0.71,3.06) | 1.76(0.86,3.60) | 1.04(0.46,2.35) | 1.96(0.96,3.98)^a^ | 0.91(0.41,2.02) | 1.74(0.84,3.61) |
|  | Marital status-adjusted OR (95%CI) | | | | | | | | | | |
|  | Triglycerides | LDL | HDL | HbA1c | Glucose | Cholesterol | MetS | Hypertension | Pre-hypertension & Hypertension | Overweight | WC |
| Housework |  |  |  |  |  |  |  |  |  |  |  |
| No | Ref. | Ref. | Ref. | Ref. | Ref. | Ref. | Ref. | Ref. | Ref. | Ref. | Ref. |
| Yes | 0.95(0.67,1.34) | 1.07(0.74,1.56) | 1.34(0.96,1.89)^a^ | 1.12(0.60,2.08) | 1.06(0.73,1.53) | 1.10(0.76,1.58) | 1.36(0.95,1.95) | 0.93(0.62,1.40) | 1.26(0.86,1.80) | 0.88(0.60,1.30) | 1.11(0.76,1.62) |
| Housework intensity | | | | | | | | | | | |
| 0-50 min/day | Ref. | Ref. | Ref. | Ref. | Ref. | Ref. | Ref. | Ref. | Ref. | Ref. | Ref. |
| 50-180min/day | 1.18(0.80,1.73) | 1.44(0.96,2.18)^a^ | 1.45(0.98,2.14)^a^ | 1.02(0.50,2.08) | 0.97(0.64,1.49) | 1.54(1.03,2.30)^b^ | 1.60(1.07,2.40)^b^ | 1.20(0.76,1.88) | 1.23(0.83,1.82) | 0.94(0.60,1.47) | 1.43(0.94,2.18)^a^ |
| ≥ 180 min/day | 1.05(0.52,2.13) | 1.43(0.69,2.99) | 1.25(0.62,2.51) | 1.46(0.48,4.47) | 1.22(0.58,2.55) | 1.45(0.70,2.99) | 2.00(0.99,4.06)^a^ | 1.23(0.55,2.73) | 2.24(1.11,4.52)^b^ | 0.96(0.43,2.13) | 1.96(0.95,4.03)^a^ |
|  | Smoking-adjusted OR (95%CI) | | | | | | | | | | |
|  | Triglycerides | LDL | HDL | HbA1c | Glucose | Cholesterol | MetS | Hypertension | Pre-hypertension & Hypertension | Overweight | WC |
| Housework |  |  |  |  |  |  |  |  |  |  |  |
| No | Ref. | Ref. | Ref. | Ref. | Ref. | Ref. | Ref. | Ref. | Ref. | Ref. | Ref. |
| Yes | 0.95(0.68,1.35) | 1.08(0.74,1.56) | 134(0.96,1.88)a | 1.09(0.59,2.04) | 1.07(0.74,1.54) | 1.11(0.77,1.59) | 1.39(0.97,1.99)a | 1.01(0.67,1.50) | 1.34(0.94,1.89) | 0.89(0.60,1.31) | 1.17(0.80,1.71) |
| Housework intensity | | | | | | | | | | | |
| 0-50 min/day | Ref. | Ref. | Ref. | Ref. | Ref. | Ref. | Ref. | Ref. | Ref. | Ref. | Ref. |
| 50-180min/day | 1.18(0.80,1.74) | 1.44(0.96,2.18)^a^ | 1.46(0.99,2.15)^a^ | 1.03(0.51,2.10) | 1.00(0.66,1.53) | 1.53(1.03,2.29)^b^ | 1.63(1.10,2.44)^b^ | 1.23(0.79,1.93) | 1.25(0.84,1.86) | 0.97(0.62,1.50) | 1.46(0.96,2.22)^a^ |
| ≥ 180 min/day | 1.04(0.51,2.11) | 1.42(0.68,2.97) | 1.27(0.63,2.56) | 1.38(0.45,4.23) | 1.30(0.62,2.71) | 1.40(0.68,2.90) | 2.07(1.02,4.20)^b^ | 1.33(0.60,2.97) | 2.35(1.16,4.74)^b^ | 0.99(0.44,2.19) | 2.03(0.99,4.18)^a^ |
|  | Drinking-adjusted OR (95%CI) | | | | | | | | | | |
|  | Triglycerides | LDL | HDL | HbA1c | Glucose | Cholesterol | MetS | Hypertension | Pre-hypertension & Hypertension | Overweight | WC |
| Housework |  |  |  |  |  |  |  |  |  |  |  |
| No | Ref. | Ref. | Ref. | Ref. | Ref. | Ref. | Ref. | Ref. | Ref. | Ref. | Ref. |
| Yes | 0.95(0.68,1.34) | 1.08(0.75,1.57) | 1.35(0.96,1.90)^a^ | 1.15(0.62,2.14) | 1.05(0.72,1.52) | 1.12(0.78,1.61) | 1.38(0.96,1.98)^a^ | 0.99(0.66,1.48) | 1.33(0.94,1.88) | 0.88(0.60,1.30) | 1.17(0.80,1.70) |
| Housework intensity | | | | | | | | | | | |
| 0-50 min/day | Ref. | Ref. | Ref. | Ref. | Ref. | Ref. | Ref. | Ref. | Ref. | Ref. | Ref. |
| 50-180min/day | 1.19(0.80,1.75) | 1.45(0.96,2.18)^a^ | 1.46(0.99,2.15)^a^ | 1.06(0.52,2.15) | 0.99(0.65,1.52) | 1.55(1.03,2.31)^b^ | 1.64(1.10,2.44)^b^ | 1.22(0.78,1.91) | 1.25(0.84,1.85) | 0.97(0.62,1.51) | 1.46(0.96,2.22)^a^ |
| ≥ 180 min/day | 1.14(0.56,2.32) | 1.43(0.68,3.00) | 1.18(0.58,2.39) | 1.42(0.46,4.35) | 1.42(0.67,2.98) | 1.50(0.73,3.10) | 2.20(1.08,4.46)^b^ | 1.29(0.58,2.87) | 2.34(1.16,4.73)^b^ | 1.08(0.49,2.41) | 2.11(1.02,4.34)^b^ |

WC: Waist Circumference; OR: Odds Ratio; CI: Confidence Interval; Ref.: Reference.

a: *p*<0.1; b: *p*<0.05; c: *p*<0.001.

Appendix II Single covariate adjusted binary logistic regression models of housework and metabolic markers for Chinese women.

|  | Education-adjusted OR (95%CI) | | | | | | | | | | |
| --- | --- | --- | --- | --- | --- | --- | --- | --- | --- | --- | --- |
|  | Triglycerides | LDL | HDL | HbA1c | Glucose | Cholesterol | MetS | Hypertension | Pre-hypertension & Hypertension | Overweight | WC |
| Housework |  |  |  |  |  |  |  |  |  |  |  |
| No | Ref. | Ref. | Ref. | Ref. | Ref. | Ref. | Ref. | Ref. | Ref. | Ref. | Ref. |
| Yes | 2.04(1.06,3.95)^b^ | 1.41(0.79,2.51) | 1.18(0.68,2.05) | 1.31(0.41,4.25) | 0.96(0.54,1.71) | 1.45(0.83,.57) | 1.32(0.72,2.41) | 0.95(0.50,1.80) | 1.05(0.60,1.82) | 1.91(0.90,4.04)^a^ | 1.04(0.63,1.74) |
| Housework intensity | | | | | | | | | | | |
| 0-50 min/day | Ref. | Ref. | Ref. | Ref. | Ref. | Ref. | Ref. | Ref. | Ref. | Ref. | Ref. |
| 50-180min/day | 1.65(1.07,2.55)^b^ | 1.38(0.93,2.06) | 1.01(0.69,1.47) | 1.00(0.47,2.13) | 1.12(0.74,1.70) | 1.29(0.88,1.90)^a^ | 1.38(0.89,2.12) | 1.00(0.63,1.59) | 1.44(0.96,2.17)a | 1.53(0.94,2.49)^a^ | 1.14(0.79,1.63) |
| ≥ 180 min/day | 1.88(1.21,2.92)^b^ | 1.29(0.86,1.93) | 1.06(0.73,1.56) | 1.20(0.56,2.57) | 1.12(0.73,1.71) | 1.23(0.83,1.82) | 1.59(1.03,2.76)^b^ | 1.12(0.70,1.78) | 1.51(0.96,2.28)a | 1.67(1.02,2.73)^b^ | 1.31(0.91,1.88) |
|  | Employment-adjusted OR (95%CI) | | | | | | | | | | |
|  | Triglycerides | LDL | HDL | HbA1c | Glucose | Cholesterol | MetS | Hypertension | Pre-hypertension & Hypertension | Overweight | WC |
| Housework |  |  |  |  |  |  |  |  |  |  |  |
| No | Ref. | Ref. | Ref. | Ref. | Ref. | Ref. | Ref. | Ref. | Ref. | Ref. | Ref. |
| Yes | 2.11(1.09,4.09)^b^ | 1.43(0.80,2.56) | 1.19(0.69,2.06) | 1.37(0.42,4.43) | 0.99(0.56,1.77) | 1.51(0.86,2.67) | 1.37(0.75,2.53) | 1.02(0.53,1.94) | 1.11(0.64,1.94) | 1.96 (0.93,4.16)^a^ | 1.09(0.65,1.82) |
| Housework intensity | | | | | | | | | | | |
| 0-50 min/day | Ref. | Ref. | Ref. | Ref. | Ref. | Ref. | Ref. | Ref. | Ref. | Ref. | Ref. |
| 50-180min/day | 1.75(1.13,2.71)^b^ | 1.44(0.96,2.14)^a^ | 1.03(0.71,1.50)^a^ | 1.07(0.50,2.29) | 1.17(0.77,1.78) | 1.36(0.93,2.01) | 1.48(0.96,2.29)^a^ | 1.10(0.69,1.74) | 1.57(1.04,2.38)^b^ | 1.59(0.98,2.60)^a^ | 1.21(0.84,1.74) |
| ≥ 180 min/day | 1.85(1.19,2.88)^b^ | 1.27(0.84,1.90) | 1.05(0.71,1.53) | 1.17(0.55,2.51) | 1.11(0.73,1.71) | 1.22(0.82,1.80) | 1.57(1.01,2.43)^b^ | 1.11(0.69,1.77) | 1.49(0.98,2.27)^a^ | 1.66(1.01,2.71)^b^ | 1.30(0.90,1.87) |
|  | Residence-adjusted OR (95%CI) | | | | | | | | | | |
|  | Triglycerides | LDL | HDL | HbA1c | Glucose | Cholesterol | MetS | Hypertension | Pre-hypertension & Hypertension | Overweight | WC |
| Housework |  |  |  |  |  |  |  |  |  |  |  |
| No | Ref. | Ref. | Ref. | Ref. | Ref. | Ref. | Ref. | Ref. | Ref. | Ref. | Ref. |
| Yes | 2.12(1.09,4.09)^b^ | 1.46(0.82,2.60) | 1.19(0.69,2.06) | 1.43(0.44,4.63) | 1.00(0.56,1.79) | 1.53(0.87,2.69) | 1.40(0.76,2.56) | 1.03(0.54,1.95) | 1.13(0.65,1.95) | 1.98(0.93,4.19)^a^ | 1.11(0.67,1.84) |
| Housework intensity | | | | | | | | | | | |
| 0-50 min/day | Ref. | Ref. | Ref. | Ref. | Ref. | Ref. | Ref. | Ref. | Ref. | Ref. | Ref. |
| 50-180min/day | 1.69(1.09,2.62)^b^ | 1.40(0.94,2.09)^a^ | 1.01(0.69,1.47) | 1.10(0.51,2.35) | 1.15(0.76,1.75) | 1.33(0.91,1.96) | 1.42(0.93,2.20) | 1.06(0.66,1.67) | 1.51(1.00,2.27)^b^ | 1.55(0.96,2.53)^a^ | 1.17(0.82,1.68) |
| ≥ 180 min/day | 1.94(1.25,3.01)^b^ | 1.31(0.88,1.97) | 1.07(0.73,1.56) | 1.23(0.58,2.65) | 1.15(0.75,1.76) | 1.27(0.86,1.87) | 1.65(1.07,2.56)^b^ | 1.16(0.73,1.85) | 1.57(1.04,2.37)^b^ | 1.69(1.03,2.76)^b^ | 1.35(0.94,1.94) |
|  | Medical insurance-adjusted OR (95%CI) | | | | | | | | | | |
|  | Triglycerides | LDL | HDL | HbA1c | Glucose | Cholesterol | MetS | Hypertension | Pre-hypertension & Hypertension | Overweight | WC |
| Housework |  |  |  |  |  |  |  |  |  |  |  |
| No | Ref. | Ref. | Ref. | Ref. | Ref. | Ref. | Ref. | Ref. | Ref. | Ref. | Ref. |
| Yes | 2.12(1.10,4.11)b | 1.44(0.81,2.57) | 1.20(0.69,2.07) | 1.40(0.43,4.52) | 0.97(0.55,1.74) | 1.51(0.86,2.65) | 1.38(0.76,2.53) | 1.01(0.53,1.92) | 1.10(0.64,1.91) | 2.05(0. .97,4.35)^a^ | 1.10(0.66,1.83) |
| Housework intensity | | | | | | | | | | | |
| 0-50 min/day | Ref. | Ref. | Ref. | Ref. | Ref. | Ref. | Ref. | Ref. | Ref. | Ref. | Ref. |
| 50-180min/day | 1.70(1.10,2.64)^b^ | 1.41(0.95,2.11)^a^ | 1.02(0.70,1.48) | 1.05(0.49,2.24) | 1.13(0.74,1.72) | 1.33(0.90,1.95) | 1.43(0.93,2.20) | 1.05(0.66,1.66) | 1.49(0.99,2.24)^a^ | 1.63(1.00,2.65)^a^ | 1.18(0.83,1.70) |
| ≥ 180 min/day | 1.92(1.24,2.97)^b^ | 1.31(0.87,1.95) | 1.07(0.73,1.56) | 1.24(0.58,2.65) | 1.13(0.74,1.73) | 1.25(0.85,1.85) | 1.63(1.05,2.52)^b^ | 1.15(0.72,1.83) | 1.54(1.02,2.32)^b^ | 1.73(1.06,2.83)^b^ | 1.34(0.93,1.93) |
|  | Marital status-adjusted OR (95%CI) | | | | | | | | | | |
|  | Triglycerides | LDL | HDL | HbA1c | Glucose | Cholesterol | MetS | Hypertension | Pre-hypertension & Hypertension | Overweight | WC |
| Housework |  |  |  |  |  |  |  |  |  |  |  |
| No | Ref. | Ref. | Ref. | Ref. | Ref. | Ref. | Ref. | Ref. | Ref. | Ref. | Ref. |
| Yes | 2.07(1.07,4.00)^b^ | 1.42(0.79,2.52) | 1.18(0.68,2.05) | 1.35(0.42,4.36) | 0.98(0.55,1.76) | 1.49(0.84,2.63) | 1.34(0.73,2.47) | 1.00(0.53,1.92) | 1.10(0.63,1.90) | 1.95(0.92,4014)^b^ | 1.08(0.65,1.79) |
| Housework intensity | | | | | | | | | | | |
| 0-50 min/day | Ref. | Ref. | Ref. | Ref. | Ref. | Ref. | Ref. | Ref. | Ref. | Ref. | Ref. |
| 50-180min/day | 1.69(1.09,2.62)^b^ | 1.40(0.94,2.09)^a^ | 1.01(0.69,1.47) | 1.03(0.48,2.19) | 1.15(0.76,1.75) | 1.33(0.91,1.96) | 1.43(0.93,2.20) | 1.06(0.67,1.67) | 1.51(1.00,2.27)^b^ | 1.55(0.96,2.53)^a^ | 1.17(0.82,1.68) |
| ≥ 180 min/day | 1.94(1.25,3.01)^b^ | 1.31(0.88,1.97) | 1.07(0.73,1.56) | 1.24(0.58,2.65) | 1.15(0.75,1.76) | 1.27(0.86,1.87) | 1.65(1.07,2.56)^b^ | 1.16(0.73,1.85) | 1.57(1.04,2.37)^b^ | 1.69(1.03,2.77)^b^ | 1.35(0.94,1.94) |
|  | Smoking-adjusted OR (95%CI) | | | | | | | | | | |
|  | Triglycerides | LDL | HDL | HbA1c | Glucose | Cholesterol | MetS | Hypertension | Pre-hypertension & Hypertension | Overweight | WC |
| Housework |  |  |  |  |  |  |  |  |  |  |  |
| No | Ref. | Ref. | Ref. | Ref. | Ref. | Ref. | Ref. | Ref. | Ref. | Ref. | Ref. |
| Yes | 2.04(1.05,3.94)^b^ | 1.41(0.79,2.51) | 1.17(0.67,2.02) | 1.32(0.41,4.27) | 0.98(0.55,1.75) | 1.49(0.85,2.63) | 1.33(0.73,2.43) | 0.99(0.52,1.87) | 1.09(0.63,1.88) | 1.92(0.91,4.06)^a^ | 1.08(0.65,1.79) |
| Housework intensity | | | | | | | | | | | |
| 0-50 min/day | Ref. | Ref. | Ref. | Ref. | Ref. | Ref. | Ref. | Ref. | Ref. | Ref. | Ref. |
| 50-180min/day | 1.66(1.08,2.57)^b^ | 1.39(0.94,2.07) | 1.00(0.69,1.46) | 1.01(0.47,2.16) | 1.14(0.75,1.74) | 1.32(0.90,1.94) | 1.40(0.91,216) | 1.04(0.66,1.65) | 1.49(0.99,2.23)^a^ | 1.55(0.95,2.52)^a^ | 1.17(0.82,1.67) |
| ≥ 180 min/day | 1.88(1.21,2.91)^b^ | 1.29(0.86,1.93) | 1.05(0.72,1.54) | 1.20(0.56,2.57) | 1.13(0.74,1.73) | 1.24(0.84,1.84) | 1.60(1.03,2.47)^b^ | 1.13(0.71,1.80) | 1.52(1.01,2.30)^b^ | 1.67(1.02,2.74)^b^ | 1.33(0.92,1.91) |
|  | Drinking-adjusted OR (95%CI) | | | | | | | | | | |
|  | Triglycerides | LDL | HDL | HbA1c | Glucose | Cholesterol | MetS | Hypertension | Pre-hypertension & Hypertension | Overweight | WC |
| Housework |  |  |  |  |  |  |  |  |  |  |  |
| No | Ref. | Ref. | Ref. | Ref. | Ref. | Ref. | Ref. | Ref. | Ref. | Ref. | Ref. |
| Yes | 2.09(1.08,4.03)^b^ | 1.43(0.80,2.54) | 1.19(0.69,2.05) | 1.37(0.42,4.43) | 0.99(0.56,1.77) | 1.50(0.85,2.64) | 1.37(0.75,2.50) | 1.02(0.54,1.93) | 1.11(0.64,1.92) | 1.96(0.92,4.14) | 1.09(0.66,1.81) |
| Housework intensity | | | | | | | | | | | |
| 0-50 min/day | Ref. | Ref. | Ref. | Ref. | Ref. | Ref. | Ref. | Ref. | Ref. | Ref. | Ref. |
| 50-180min/day | 1.69(1.09,2.61)^b^ | 1.40(0.94,2.08)^a^ | 1.01(0.69,1.47) | 1.04(0.48,2.23) | 1.15(0.76,1.75) | 1.32(0.90,1.94) | 1.43(0.93,2.20) | 1.06(0.67,1.68) | 1.50(1.00,2.26)^a^ | 1.57(0.96,2.55)^a^ | 1.18(0.82,1.68) |
| ≥ 180 min/day | 1.92(1.24,2.98)^b^ | 1.30(0.87,1.94) | 1.07(0.73,1.56) | 1.26(0.59,2.71) | 1.15(0.75,1.76) | 1.25(0.84,1.84) | 1.65(1.07,2.56)^b^ | 1.17(0.73,1.86) | 1.55(1.03,2.34)^b^ | 1.71(1.05,2.79)^b^ | 1.35(0.94,1.94) |

WC: Waist Circumference; OR: Odds Ratio; CI: Confidence Interval; Ref.: Reference.

a: *p*<0.1; b: *p*<0.05; c: *p*<0.001.
